# Frequency of typical and atypical computed tomography findings of COVID-19 and their effect on hospitalization

**DOI:** 10.14744/nci.2021.24865

**Published:** 2021-12-29

**Authors:** Mahmut Corapli, Ercan Cil, Cemil Oktay, Haci Taner Bulut, Gokhan Corapli

**Affiliations:** 1. Department of Radiology, Adiyaman Training and Research Hospital, Adiyaman, Turkey; 2. Department of Chest Diseases, Adiyaman University Faculty of Medicine, Adiyaman, Turkey; 3. Department of Radiology, Adiyaman University Faculty of Medicine, Adiyaman, Turkey; 4. Department of Chest Diseases, Yuksekova State Hospital, Hakkari, Turkey

**Keywords:** Atypical findings, computed tomography, COVID-19, ground-glass opacity

## Abstract

**Objective:**

This study aimed to determine the frequency of typical and atypical computed tomography (CT) findings of COVID-19 and their effect on hospitalization.

**Methods:**

We retrospectively assessed 414 patients who were diagnosed with COVID-19 by real-time reverse transcription-polymerase chain reaction and who had lung involvement in their admission chest CT. We evaluated the frequency of typical and atypical chest CT findings and analyzed the relationship between typical and atypical findings of COVID-19 in patients treated in ambulatory versus inpatient settings.

**Results:**

Ground-glass opacities were the most common typical finding of COVID-19 chest CT scans. The frequencies of other typical findings, including consolidation, air bronchogram, pulmonary vascular enlargement (PVE), airway changes, crazy paving pattern, and reticular pattern, were similar to those reported in the literature. Atypical findings were less common and found at varying frequencies. Crazy paving pattern, air bronchogram, reticular pattern, and PVE were significantly more common in hospitalized patients (p<0.001). The frequencies of other typical and atypical findings were not significantly different between ambulatory and hospitalized patients.

**Conclusion:**

Increased recognition of typical and atypical findings of COVID-19 and their frequencies, as well as knowledge of admission chest CT findings that are associated with hospitalization, will facilitate medical care during the pandemic.

**I**n December 2019, an outbreak of pneumonia of unknown origin was reported in Wuhan, China. Subsequent studies identified a novel coronavirus, SARS-CoV-2, as the etiological agent of this viral infection. Through person-to-person transmission, the disease rapidly spread around the world. In March 2020, the World Health Organization declared the coronavirus outbreak as a pandemic [1, 2].

Real-time reverse transcription-polymerase chain reaction (RT-PCR) is a commonly used method for detecting SARS-CoV-2 infections due to being fast, easy to use, and practical. In addition, computed tomography (CT) of the chest is easily applied for patients with suspected COVID-19, especially in regions affected by the pandemic, and is very valuable in supporting the diagnosis. According to recent studies, chest CT is more sensitive than RT-PCR in determining COVID-19 and its prognosis [3, 4].

Typical chest CT findings in COVID-19 include bilateral, mostly peripheral multiple ground-glass opacities (GGOs). Despite being common in COVID-19 pneumonia, chest CT findings may vary between individuals [5–7]. Besides GGOs, other typical findings of COVID-19 include consolidations, air bronchogram, pulmonary vascular enlargement (PVE), airway changes, crazy paving pattern, and reticular pattern (Fig. 1) [8]. Atypical chest CT findings of COVID-19 include pleural thickening and/or effusion, nodules, reversed halo signs, lymphadenopathy (LAP), pericardial effusion, subpleural lines, subpleural sparing, spider web signs, air bubble signs, tree-in-bud signs, central and/or peribronchovascular involvement, isolated upper lobe involvement, and isolated lobar consolidation (Fig. 2, 3) [2, 8–10]. Having a comprehensive knowledge of the typical and atypical chest CT findings of COVID-19 will help to reduce false negatives and improve the management of the outbreak [11]. In this study, we aimed to establish the frequency of typical and atypical CT findings in our hospital and their effect on hospitalization.

**Figure 1.**
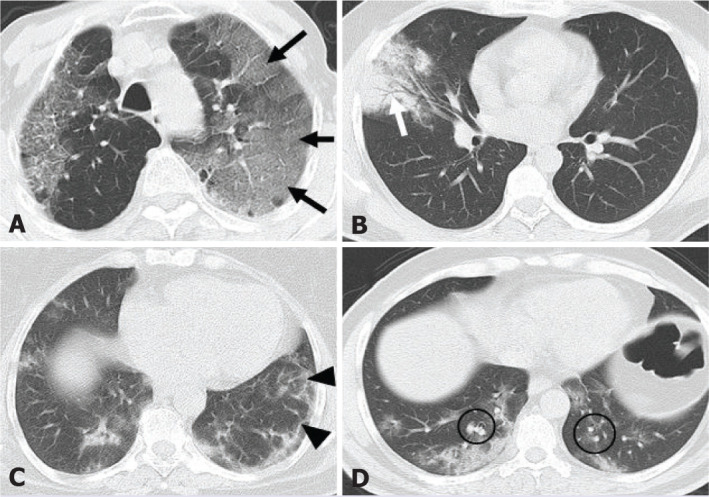
The axial section of the chest computed tomography scan shows typical findings **(A)** ground glass opacity, crazy paving pattern (black arrows); **(B)** consolidation, air bronchogram (white arrow); **(C)** reticular pattern (black arrow-heads); **(D)** pulmonary vascular enlargement (black circle).

**Figure 2.**
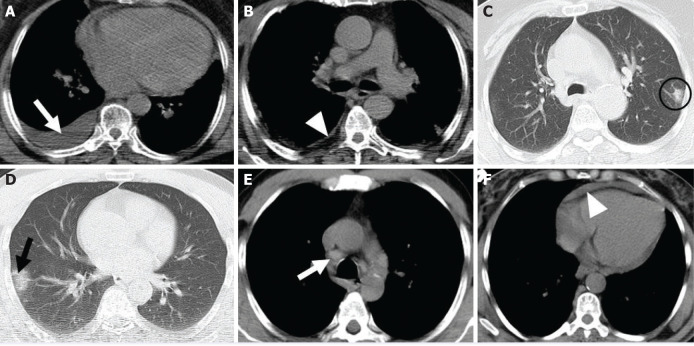
The axial section of the chest computed tomography scan shows atypical findings **(A)** pleural effusion (white arrow), **(B)** pleural thickening (white arrow-head), **(C)** nodule (black circle), **(D)** reversed halo sign (black arrow), **(E)** mediastinal lymph nodes (white arrow), **(F)** pericardial effusion (white arrow-head).

**Figure 3.**
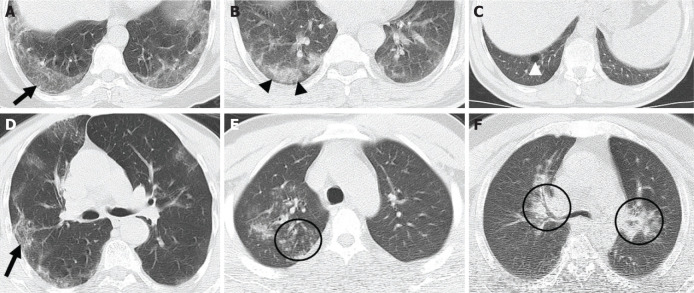
The axial section of the chest computed tomography scan shows atypical findings **(A)** subpleural line (black arrow), **(B)** subpleural sparing (black arrow-heads), **(C)** air-bubble sign (white arrow-head), **(D)** spider web sign (black arrow), **(E)** tree-in-bud (black circle), **(F)** central-peribronchovascular involvement (black circle).

## Materials and Methods

This study was approved by Adiyaman University Non-Interventional Clinical Research Ethics Board (approval code: 2021/01-19) and the Ministry of Health of the Republic of Turkey (approval date: 25.12.2020).

### Study Population

We retrospectively assessed records of the patients who were diagnosed with COVID-19 by nasopharyngeal and oropharyngeal RT-PCR between September 2020 and January 2021.

The inclusion criteria included:

1. Being diagnosed with COVID-19 by clinical examination and RT-PCR
2. Unenhanced chest CT obtained at the time of admission
3. Exclusion of other causes of pneumonia.

The exclusion criteria included:

1. COVID-19 diagnosis not being confirmed by RT-PCR
2. Having received treatment before CT imaging
3. Lack of unenhanced admission chest CT images
4. Having a known lung disease
5. Having a non-diagnostic chest CT image with artifacts
6. Patients with non-COVID-19 pneumonia and/or findings suggestive of co-infection.

**Highlight key points**

• CT of the chest is easily applied for patients with suspected COVID-19.
• The most common typical finding is bilateral patchy peripheral ground-glass opacity on COVID-19 pneumonia.
• In addition to ground-glass opacity, less frequent other typical findings are common, while atypical findings are seen at varying rates.
• Among the typical chest CT findings, crazy paving pattern, air bronchogram, reticular pattern, and PVE were significantly more common in hospitalized patients.
• The recognition of uncommon atypical findings together with the more common typical findings will facilitate clinical practice during the pandemic.

### CT Protocol

All examinations were performed with a 16-slice multidetector CT scanner (MX16, Philips Medical System, Koninklijke, Netherlands). CT images were obtained in the supine position during deep inspiration. The examinations were carried out with a single CT device reserved for the patients with suspected COVID-19 in our hospital within the scope of the precautions taken with the arrival of the pandemic to our country. And according to these precautions, the CT scanner was disinfected after each exam. Scanning and reconstruction parameters were as follows: beam collimation, 16×0.75 mm; rotation time, 0.75 s; slice thickness, 1 mm; slice reconstruction, 1 mm; tube voltage, 90–120 kV; effective tube current-time product, 50–110 mAs. CT images were acquired from lung apex to lung base. The field of view was 250–300 mm.

### Image Interpretation

The images were interpreted using the hospital database (Oracle Database V1.10.43.134). All CT images were independently evaluated by two radiologists, one with 6 years (M.Ç.) and one with 3 years (C.O.) of experience in radiology. In the event of a discrepancy between these two observers, a third observer with 16 years of experience in radiology (H.T.B.) reviewed the images to reach an agreement.

### Clinical Examination

The indication for hospitalization was made according to the criteria defined by the COVID-19 diagnosis and treatment guidelines of the Ministry of Health of the Republic of Turkey. According to this guideline, a patient who meets at least one of the following criteria was hospitalized [12]:

1. Mild-to-moderate pneumonia (bilateral <50% radiological involvement) AND respiratory rate ≥24/min and SpO_2_ ≤93%
2. Mild-to-moderate pneumonia AND poor prognosis criteria in admission blood tests (lymphocyte count <800/μL or serum CRP values >10 times upper limit of normal or ferritin >500 ng/mL or D-dimer >1000 ng/mL)
3. Severe pneumonia (altered state of consciousness, respiratory distress, respiratory rate ≥30/min, SpO_2_ ≤90% in room air, widespread (>50%) bilateral lung involvement)
4. Hypotension (<65 mmHg)
5. Tachycardia (>100 bpm)
6. Sepsis
7. Septic shock
8. Myocarditis
9. Acute coronary syndrome
10. Arrhythmia
11. Acute kidney injury.

### Statistical Analysis

Statistical analyses were carried out using Statistical Package for Social Sciences program version 23.0 Inc. Chicago, IL, USA. All variables were categorical and descriptive statistics for categorical variables were given as numbers (n) and percentages. Statistical analysis was performed to compare typical and atypical tomography findings in ambulatory and inpatient patients. Categorical variables were compared using Pearson’s Chi-squared test or Fisher’s exact test. Significance level was considered p<0.05.

## Results

About 2405 patients with RT-PCR positivity were evaluated and among them, 414 patients who met the inclusion and exclusion criteria were enrolled for this study. Of the patients, 182 (44%) were female, 232 (56%) were male, and the average age was 52.2±15.1 years (range: 18–88 years).

The frequencies of typical chest CT findings were as follows: GGOs, 388 (93.7%); consolidation, 218 (52.6%); GGOs+consolidation, 194 (46.8%); crazy paving, 213 (51.4%); air bronchogram, 153 (36.9%); airway changes, 26 (6%); reticular pattern, 176 (42.5%); and PVE, 141 (34%) (Table 1).

**Table 1.** The reported frequency of the typical chest computed tomography findings

	Current study	Zarifian et al. (metaanalysis) [16]	Zhao et al. [17]	Bai et al. [18]	Zhang et al. [19]	Li et al. [20]	Wu et al. [21]	Bernheim et al. [2]
Number of patients	(n=414) %	(n=9907) %	(n=101) %	(n=219) %	(n=120) %	(n=56) %	(n=80) %	(n=121) %
GGO	93.7	6224–4799 (77.1%)	86.1	91	89	80.4	91	75.2
Consolidation	52.6	4397–1560 (35.5%)	43.6	69	52	21.4	63	42.9
GGO+Consolidation	46.8	385–146 (38%)	64.4	64	–	76.8	–	41
Crazy paving	51.4	–	–	5	25	44.6	29	5
Air bronchogram	36.9	1953–800 (41%)	–	14	20	73.2	–	–
Airway changes	6	994–136 (13.6%)	–	9	12	–	11	13
Reticular pattern	42.5	2667–1232 (46.2%)	48.5	56	18	53.6	20	7
PVE	34	–	71.3	59	–	–	–	–

GGO: Ground glass opacity; PVE: Pulmonary vascular enlargement.

The frequencies of atypical chest CT findings were as follows: pleural thickening and/or effusion, 70 (16.9%); nodules, 24 (5.7%); reversed halo signs, 5 (1.2%); LAP, 9 (2.1%); pericardial effusion, 7 (1.6%); subpleural lines, 4 (0.9%); subpleural sparing, 14 (3.3%); spider web signs, 12 (2.8%); air bubble signs, 13 (3.1%); tree-in-bud signs, 4 (0.9%); central and/or peribronchovascular involvement, 8 (1.9%); and isolated upper lobe involvement, 16 (3.8%) (Table 2).

**Table 2.** The reported frequency of the atypical chest computed tomography findings

	Current study	Zarifian et al. (metaanalysis) [16]	Li et al. [20]	Wu et al. [21]	Pakdemirli et al. [22]	Wang et al. [23]	Tabatabaei et al. [24]	Werberich et al. [25]
Number of patients	(n=414)	(n=9907)	(n=83)	(n=80)	(n=18)	(n=93)	(n=120)	(n=48)
Pleural thickening and/or effusion	16.9	5345–741 (13.8%)	8.4	6	88	31.1	16.6	4.2
Nodule	5.7	2582–338 (13.1%)	7.2	–	61	18.3	25	4.2
Reversed halo sign	1.2	–	–	–	0	15.1	11.6	4.2
LAP	2.1	3197–165 (5.1%)	8.4	4	17	6.5	0	4.2
Pericardial effusion	1.6	–	4.8	5	–	–	–	6.2
Subpleural line	0.9	–	20.5	20	61	–	–	14.6
Subpleural sparing	3.3	–	–	–	33	–	23.3	6.2
Spider web sign	2.8	–	25.3	25	–	–	–	–
Air bubble sign	13 (3.1%)	–	–	–	–	12.9	–	37.5
Tree-in-bud	4 (0.9%)	–	–	–	6	3.2	0	–
Central and/or	1.9	2160–131 (6.1%)	–	4	11	–		2.1
peribronchovascular involvement								
Isolated upper lobe involvement	3.8	–	–	–	0	–	–	10.4

LAP: Lymphadenopathy.

Of the subjects, 196 (47.3%) and 218 (52.7%) were treated and followed in ambulatory and inpatient settings, respectively. Seven (3.2%) patients died during inpatient treatment. Among the typical chest CT findings, crazy paving pattern, air bronchogram, reticular pattern, and PVE were significantly more common in hospitalized patients (p<0.001). The frequencies of other typical and atypical findings were not significantly different between ambulatory and hospitalized patients (Table 3).

**Table 3.** The frequency of typical and atypical chest computed tomography findings in outpatients and inpatients

	Outpatients (n=196) (%)	Inpatients (n=218) (%)	p
Typical Findings			
GGO	92.3	95	0.314
Consolidation	50.5	54.6	0.431
GGO+Consolidation	43.4	50	0.200
Crazy paving	41.8	60.1	**<0.001**
Air bronchogram	27.6	45.4	**<0.001**
Airway changes	6.1	12 (5.5	0.788
Reticular pattern	30.1	53.7	**<0.001**
PVE	25	42.2	**<0.001**
Atypical findings			
Pleural thickening	13.3	14.7	0.777
and/or effusion
Nodule	5.1	6.4	0.675
Reversed halo sign	2	0.5	0.194
LAP	3.1	1.4	0.318
Pericardial effusion	1	2.3	0.454
Subpleural line	1	0.9	0.647
Subpleural sparing	5.1	1.8	0.058
Spider web sign	3.1	2.8	0.540
Air bubble sign	3.6	2.8	0.78
Tree-in-bud	1	0.9	0.647
Central and/or peribronchovascular involvement	2	1.8	0.578
Isolated upper lobe involvement	5.6	2.3	0.080

GGO: Ground glass opacity; PVE: Pulmonary vascular enlargement; LAP: Lymphadenopathy.

## Discussion

Chest CT plays an important role in the diagnosis of the patients with suspected COVID-19, particularly when the RT-PCR test is negative. This test may show a false negative result, especially in the initial phase of symptoms [13, 14]. And as stated in the COVID-19 diagnosis and treatment guidelines of the Ministry of Health of the Republic of Turkey, showing lung involvement makes a significant contribution to the determination of the treatment process [12]. Together with typical findings, the presence and increased recognition of atypical findings will facilitate both the diagnosis of COVID-19 with chest CT and the disease management [3, 15]. In this study, we identified the frequency of typical and atypical CT findings and their effect on hospitalization.

In our study, the most common typical finding was GGO (93.7%). This finding is consistent with the literature, where GGOs are reported in 75%–91% of COVID-19 patients. In a meta-analysis by Zarifian et al. [16] that included data from 103 studies, 4799 of 6224 patients (77.1%) had GGOs. The frequencies of consolidation, GGOs+consolidation, air bronchogram, airway changes, and reticular pattern reported in the same study are consistent with our results. In our study, we also evaluated the frequencies of crazy paving pattern and PVE. The literature reports that the prevalence of crazy paving pattern ranges between 5% and 44% in COVID-19 patients. In our study, this rate was 51.4%. PVE was found in 59%–71% of the patients in the literature and in 34% of our patients [2, 16–21]. In the literature, the prevalence of typical findings is mostly similar, with small differences that may be ascribed to variances in study populations.

In our study, pleural thickening and/or effusion and nodules were the most common atypical chest CT findings, with prevalence of 16.9% and 5.7%, respectively. Our results are generally consistent with those reported in the literature, with the exception of a study by Pakdemirli et al. [22] reporting the incidence of pleural thickening and/or effusion and nodules to be 88% and 61%, respectively. This discrepancy may be due to the small sample size (n=18) in the study by Pakdemirli et al.

Compared to typical findings, the prevalence of atypical findings reported in the literature is highly variable. In our study, the prevalences of reversed halo signs, LAP, pericardial effusion, subpleural lines, subpleural sparing, spider web signs, air bubble signs, tree-in-bud signs, central and/or peribronchovascular involvement, and isolated upper lobe involvement were 1.2%, 2.1%, 1.6%, 0.9%, 3.3%, 2.8%, 3.1%, 0.9%, 1.9%, and 3.8%, respectively. Atypical findings evaluated in the literature vary. These differences may stem from a lack of a clear definition. Another reason may be the differences in study populations and working conditions during the pandemic. For patients with COVID-19, the literature reports reversed halo signs in 0–15%, LAP in 0–17%, pericardial effusion in 4–6%, subpleural lines in 14–61%, subpleural sparing in 6–33%, spider web signs in 25%, air bubble signs in 12–37%, tree-in-bud signs in 0–6%, central and/or peribronchovascular involvement in 2–11%, and isolated upper lobe involvement in 0–10% of individuals [15, 16, 21–25].

The literature on the effect of typical and atypical chest CT findings of the COVID-19 on hospitalization is highly limited. Emara et al. [26] investigated the impact of findings on intensive care unit (ICU) admission and mortality. They found that pleural effusion and GGOs were significantly associated with ICU admission and mechanical ventilation, but they did not report the impact of typical and atypical findings on mortality. In our study, we found that crazy paving pattern, air bronchogram, reticular pattern, and PVE were significantly associated with hospitalization. Other typical and atypical findings were not significantly associated with hospitalization. In our study, we were unable to assess ICU admission and mechanical ventilation since our primary objective was to investigate the impact of admission chest CT findings on hospitalization and to establish whether chest CT findings, as well as other clinical criteria [12], contribute to the decision of hospitalization.

The high percentage of hospitalized patients in our study is due to including patients with pulmonary involvement in their admission chest CT images. We excluded patients without pulmonary involvement regardless of a positive RT-PCR test. Our mortality rate (1.6%) is similar to those reported by the Ministry of Health of the Republic of Turkey [27]. The limitations of our study include not being able to investigate the association between chest CT findings and mortality due to the small number of deaths, the retrospective and single-center design, and having a small number of the patients with atypical findings. Further studies with larger samples are needed to clarify the impact of atypical findings on COVID-19 prognosis. Furthermore, we did not include CT images from during and after treatment; therefore, we did not evaluate atypical findings that may occur during or after treatment. Finally, exclusion of bacterial infections accompanying COVID-19 infection with only RT-PCR test and initial thoracic CT findings may be insufficient to exclude all co-infections. However, it is known that the bacterial co-infection rate is low in COVID-19 pneumonia [28, 29]. However, it should be kept in mind that this may affect the frequency of atypical findings, while evaluating the results of the study.

### Conclusion

The recognition of uncommon atypical findings together with the more common typical findings will help to prevent false negatives in radiology reports and the diagnosis of COVID 19. When evaluating admission chest CT findings, knowing the clinical criteria and chest CT findings that will affect hospitalization will facilitate clinical practice during the pandemic.
